# Hemorrhagic Malarial Fever

**Published:** 1882-04

**Authors:** R. M. Brown

**Affiliations:** Ga.


### HEMORRHAGIC MALARIAL FEVER.

By R. M. Brown, M.D.,——, Ga.

Not having seen any mention of this disease in the journals, I wish to present an outline of my plan of treatment. As it is a disease, which is very often fatal, I will be glad if any one would suggest his own plan. It is I think essentially of malarial origin. Practicing in a district of this kind, I have had several cases some of which died, and others recovered. My friend, Dr. W. R. Robinson, who practiced here for several years and was very successful in the treatment of this affection, has given me some valuable information. The disease resembles yellow fever in some of its phases. The patient suffers a prodromic stage of several days, viz: a dull, achy feeling; then follows a succession of severe rigors which are succeeded by fever of the remittent type; there are no intermissions. Another point is, that even while the rigor is present, the thermometer shows an elevated temperature. There is constant nausea and frequent, violent vomiting. Bowels usually constipated, yet there is a desire to go to stool. The urine is scanty, and great difficulty is experienced in passing it, as there seems to be atony of the bladder. Urine is very dark, tests showing the presence of blood corpuscles; from this the disease derives its name. Pulse usually full, but slugglish. In some cases the vomited matter is of an inky hue. Skin is a very deep yellow, the whites of the eyes partaking of same hue, eyes are sunken, and patient has a death-like appearance. The duration of the attack is from ten to fifteen days.

*Treatment.*—During the first period, endeavor to relieve the severity of the rigors by means of hot mustard baths, hot irons to the feet, and friction over the body. In the febrile stage, use enemata of sulph. quinine 40 grs. at each injection. I use for this purpose, a six ounce hard rubber syringe. The patient should be well cinchonized; for this purpose use the syringe, as he cannot even retain water on the stomach. I give two-drop doses of digitalis, every two or three hours; and watch the patient closely. Fluid ext. of gelsemium acts well in producing a free flow of urine; it also reduces temperature. If the patient is restless give chloral in 20 gr. doses, or use morphia hypodermically.

